# Progress of Surgery

**Published:** 1901-02-02

**Authors:** 


### Progress of Surgery.

### SURGERY OF THE INTESTINES.

**Duodenal Ulcer.**—Weir[1] states that these ulcers are generally situated within one and a half inches of the pylorus, and are most frequently encountered on the anterior wall. Of one hundred and seventy-six cases of duodenal ulcers, one hundred and thirty-four were in men and thirty in women. This has been explained to be due to the rougher food, and, in men, to their addiction to alcohol and tobacco, which intensify the acidity of the gastric juice, and thus acts unduly on the glandular mucous membrane of the duodenum which is not protected until the alkaline bile and pancreatic fluid is poured into the intestine at the opening of Vater. The following contrasting symptoms have been grouped by Vonwyl as an aid to the differential diagnosis of gastric and duodenal ulcers:—Gastric ulcer: (1) More frequent in women, twenty-fifth to fiftieth year; (2) pain promptly after eating; (3) relieved by vomiting; (4) frequent biliary, mucous, and food vomiting; (5) marked dyspeptic symptoms; (6) frequent bloody vomiting; (7) more seldom bloody stools. Duodenal ulcer: (1) occurs most frequently in males; (2) pain in right hypochondrium or to right of parasternal line; (3) comes on two to four hours after meals; (4) no relief by vomiting—latter not frequent; (5) bloody stools (melœna or bright blood), more common than bloody vomiting; (6) if jaundice is present this would contribute to the diagnosis. The incison to be advised is in or along the edge of the rectus muscle four to six inches in length and starting rather low down on the abdominal wall, so as not to reach much above the liver edge but to extend far enough downwards to get easily below the transverse colon, which might be necessary. Supplementing this incision in its upper third another transverse one in the skin to the left or toward the median line is advisable. This will permit the cross-division of the fasciæ covering the right rectus muscle, which latter is in turn to be pulled to the left, when its posterior sheath with the peritoneum is also to be divided. This gives a satisfactory and largely increased exposure of the parts beneath, and the subsequent suture of the upper and lower sheath of the rectus with the full replacement of the muscle adequately restores the integrity of the abdominal wall. As regards the fifty-one cases which Weir has collected the ulcer was recognised and closed in twenty-five; in thirteen operation was performed after thirty hours delay and all were fatal; twelve patients were operated upon immediately, of whom eight recovered.[2] Bolton,[3] quoting Boas, groups the symptoms of duodenal ulcer under the following heads:—(1) Sensitive points—correspond to seat of pain in parasternal line, and are below the gall bladder; (2) vomiting, probably reflex, occurs after prolonged and severe pain, and is present in 17 per cent. of cases; (3) intestinal hæmorrhage and hæmatemesis are present in one-third of the cases to a marked degree; (4) composition of the stomach contents as to the amount of acidity, gives no reliable evidence; (5) jaundice is not significant.

**Intussusception.**—Brunner[4] mentions several cases of intussusception treated by resection. A most unique feature in one case was the presence of an accessory pancreas adherent to the blind end of a Meckel's diverticulum which started the invagination. Another case showing the advantage of early operation is recorded by Lupton,[5] who successfully performed laparotomy for this condition on an infant ten months old; and Ray[6] also refers to four cases of acute ileo-cæcal intussusception in which laparotomy was performed. In three of these last cases reduction was effected after laparotomy and resulted in two recoveries and one death. In the remaining case the intussusception was irreducible, and the entire intussusception was excised and end-to-end anastomosis was performed by the use of Murphy's button, but this case terminated fatally. In all four of Ray's cases either a more or less definite tumour or the *signe de Dance* (lessened resistance to palpation over the right iliac fossa) could be made out, and in all the cases an acute onset was speedily followed by the passage of blood and mucus per anum.

**Meckel's Diverticulum.**—Inflammation of Meckel's diverticulum may simulate appendicitis. Such cases are reported by Nicolson[7] and Erdmann.[8] As there is no absolute means of knowing when a Meckel's diverticulum is present in the abdomen, it is far more reasonable to expect one suffering from intestinal paresis and peritonitis to be suffering from involvement of the appendix; and when one remembers that the usual location of these diverticula is within 3 feet of the cæcum, no great amount of suggestion is necessary to show the effect symptomatically of an inflammation or involvement of a viscus in such proximity to the appendix. Naturally, the conditions of obstruction rapidly supervene upon those of sympathetic involvement, and thus the diagnosis of obstruction from some other cause than appendicitis is more readily made except where profound sepsis is present.

[1] Med. Rec., May 5, 1900. [2] Lancet, June 30, 1900. [3] Med. Rec., March 24, 1900. [4] Annals of Surgery, June 1900. [5] Lancet, Aug. 18. 1900. [6] Ibid., Sept. 29, 1900. [7] New York Med. Jour., June 23, 1900. [8] Med. Rec., Oct. 27, 1900.

